# Beach Pollution Effects on Health and Productivity in California

**DOI:** 10.3390/ijerph16111987

**Published:** 2019-06-04

**Authors:** Jingjing Li, Xiaohan Zhang

**Affiliations:** 1 Department of Geosciences and Environment, California State University Los Angeles, Los Angeles, CA 90032, USA; jli104@calstatela.edu; 2 Department of Economics and Statistics, California State University Los Angeles, Los Angeles, CA 90032, USA

**Keywords:** beach pollution, fecal contamination, economic loss, health burden, productivity

## Abstract

The United States (U.S.) Clean Water Act triggered over $1 trillion in investments in water pollution abatement. However, treated sewage discharge and untreated runoff water that are contaminated by fecal matter are discharged into California beach waters daily. Warnings are posted to thwart the public from contacting polluted coastal water, according to the California Code of Regulations (CCR). This paper evaluated the current policy by empirically examining the productivity loss, in the form of sick leave, which is caused by fecal-contaminated water along the California coast under the CCR. The findings of this study showed that Californians suffer productivity losses in the amount of 3.56 million sick leave days per year due to recreational beach water pollution. This paper also empirically examined the pollution-to-sickness graph that Cabelli’s classic study theoretically proposed. The results of the research assure that the existing water quality thresholds are still reasonably safe and appropriate, despite the thresholds being based on studies from the 1950s. The weakness of the CCR lies in its poor enforcement or compliance. Better compliance, in terms of posting pollution advisories and increasing public awareness regarding beach pollution effects on health, would lead to a significant decrease in sick leaves and a corresponding increase in productivity. Therefore, this study advocates for stronger enforcement by displaying pollution advisories and better public awareness of beach pollution effects on health.

## 1. Introduction

California beaches attract 23 million residents and 150 million tourists each year [1,2]. However, large volumes of treated sewage discharge and polluted runoff water flow into the California coastline through storm drains, which are adjacent to many frequently visited California beaches. As the runoff water travels across the land surface and flows through watersheds down to the coast, the runoff water accumulates fecal contamination along the way, which originates from various sources, such as animal waste and leaky sewage pipes [3]. Treated sewage discharge also has a significant amount of pathogens, despite its treatment [4]. As a result, substantial amounts of human and animal fecal matter is frequently released into the marine coastal waters. Such urban runoff pollution has a strong negative impact on the water quality of California’s coastal water, estuaries, and bays [5,6]. 

Epidemiological studies have suggested that exposure to recreational waters that are polluted by animal and human waste may result in illnesses, which include respiratory diseases, gastrointestinal illnesses (GI), and skin, eye, and ear infections [7,8,9,10,11,12,13]. For instance, Fleisher et al. [7] surveyed 1216 UK participants consisting of swimmers and non-swimmers during the summers of 1989–1992. Their study found an association between swimming in contaminated marine waters and gastroenteritis, respiratory disease, and eye and ear infections. Wade et al. [8] examined three beaches in Mississippi, Alabama, and Rhode Island close to treated sewage discharges and discovered that high levels of fecal matter can lead to 5% swimmers suffering from GI track symptoms. Swimming, bathing, or any water activities done in feces polluted water may lead to significant health hazards, reduced productivity, and economic loss [14,15,16]. One study by Given et al. [14] estimated that 627,800–1,497,200 excess gastrointestinal illnesses occurred each year due to swimming in polluted coastal water in Los Angeles and Orange counties, which caused an economic loss of $21–$51 million dollars. Another study by DeFlorio-Baker et al. [15] estimated 90 million recreational waterborne illnesses in the United States and an economic burden of $2.2 to $3.7 billion annually, which included the costs related to lost productivity, doctor visits, medication expenses, and so on. 

In addition, several papers examined the health effects of visiting polluted California beaches [17,18,19]. For example, Turbow et al. and Dwight et al. both studied two beaches in Orange County that have more than 5.5 million visits each year. Their study estimated that nearly 37,000 GI episodes and 38,000 other illnesses per year resulted from the fecal contamination of these two beaches’ waters, which resulted in losses of $3.3 million dollars [18,19]. Brinks et al. [17] estimated 689,000 to 4,003,000 GI episodes and 693,000 respiratory episodes occur due to polluted coastal waters each year in Southern California. However, none of these studies collected empirical data to examine the pollution-to-sickness relationship. Instead, these studies simulated the disease numbers while using the pollution-to-disease statistics that were generated in [20] or [21]. 

Swimming beaches are required to be monitored for fecal bacteria to protect the swimmers from contacting feces contaminated water, according to the California State Legislation Assembly Bill 411 (AB 411) [22]. The California Code of Regulations (CCR) for Ocean Beaches and Ocean Water-Contact Sports Area pursuant to AB 411 provide the minimum protective thresholds for total coliform, fecal coliform, and enterococci (ENT) bacteria [23]. The CCR states that the magnitude of ENT in a single sample, which is also known as the Statistical Threshold Value (STV), from each sampling location should not exceed 104 cfu/100 mL [18]. Additionally, the Geometric Mean (GM) of ENT during any monthly sampling period should not be greater than 35 cfu/100 mL [23]. Here, the ENT GM criterion of 35 cfu/100 mL corresponds to the mean illness rate of 36 NEEAR-GI (NGI) per 1000 primary contact recreators [24]. Swimming advisories for the public beaches are issued by the county health care agencies when either of the water quality regulations is violated. The effectiveness of this policy and the thresholds are constantly called into question [17,20,25]. The United States (U.S.) Environmental Protection Agency (USEPA) referred to the study upon which the standards were based as “far from definitive” [25]. Moreover, the initial density was arbitrarily cut in half to 200 fecal coliforms per 100 mL in the 1960s and was converted coliform into ENT standards in 1986 without further research on ENT [25]. Therefore, it is crucial to empirically examine whether the policy effectively protects the health of beach visitors.

In contrast to the previous literature, this study aims to empirically examine the fecal pollution-to-sickness relationship in coastal California under the current regulations. Sickness is considered to be a type of negative health shock and it may cause productivity loss among workers. This productivity loss is measured by the number of leaves reported by participants of the Current Population Survey (CPS). This study exploited arguably exogenous variations in coastal water quality and the policy thresholds, and estimated the increase in the sick leaves among the local working population that resulted from beach water fecal contamination under the current policy. In addition, this study provided the first empirical version of the theoretical pollution-to-sickness curve in Cabelli’s benchmark study [20]. This information may assist in the determination of future beach water quality standards.

## 2. Materials and Methods

### 2.1. Materials

#### 2.1.1. CEDEN Water Pollution Data

**Figure 1:**
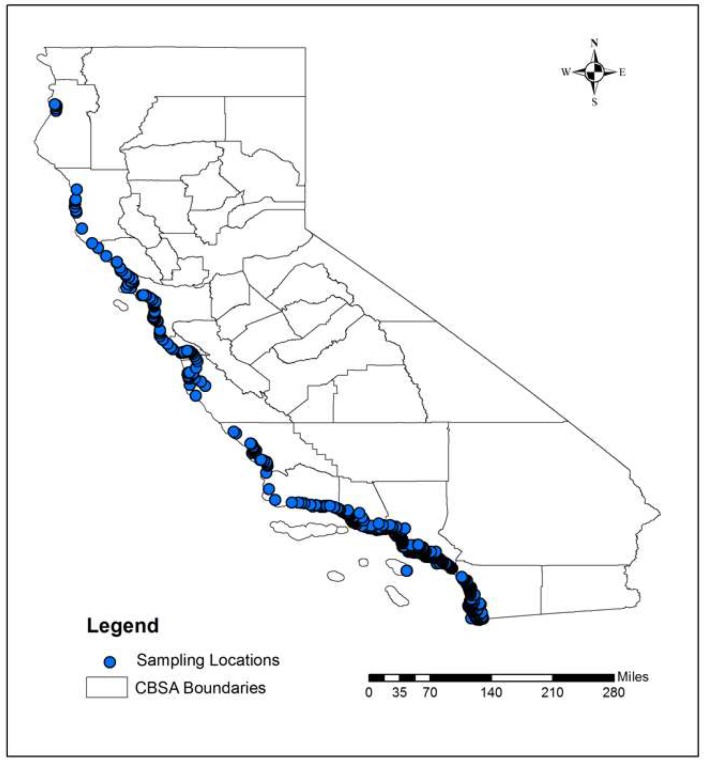
Map of Sampling Locations in California. The blue dots indicate the sampling locations obtained from California Environmental Data Exchange Network (CEDEN). The Core-Based Statistical Area (CBSA) boundaries are from the US Census in 2010.

The ENT data that were used in this study were obtained from the California Environmental Data Exchange Network (CEDEN). The State Water Resources Control Board manages the CEDEN to record the surface water quality test results [26,27]. This data portal contains the same water testing results that are used to determine the posting of the health advisories by the county health care agencies. The CEDEN documents the time-stamped surface water testing results at the chosen sampling locations near the beaches. These sampling locations were strategically chosen to represent the water quality of the coastline. Such sampling locations must be within the Core-Based Statistical Area (CBSA) and have valid ENT observations during the study period from 2004 to 2013. These points were collected from the CEDEN data portal. Figure 1 shows the spatial distribution of the sampling locations along the coast, where the ENT data were collected for this study. Some of the coastal areas do not have the sampling points, as seen in Figure 1. This could be due to the fact that (1) ENT observations were not sampled throughout the study time, (2) ENT observations were missing or not valid during this period, or (3) ENT observations were not within the studied CBSAs. 

**Figure 2:**
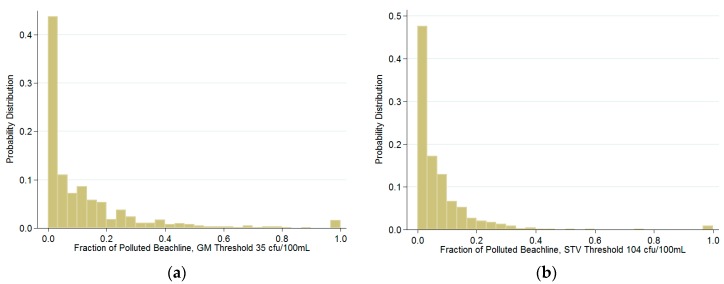
Probability of Fecal Matter Contamination at California Coasts. This figure depicts the probability distribution of the polluted beachline variable, which ranged from 0 to 1 (or 0% to 100%). The enterococci (ENT) pollution levels were from CEDEN 2004–2013. Fecal-contaminated was defined as the monthly ENT index higher than 35 cfu/100 mL in (**a**), and daily ENT index higher than 104 cfu/100 mL in (**b**).

Table 1 summarizes the data that were used for analysis. The fraction of polluted beachlines that lie within a Core-Based Statistical Area (CBSA) represent the probabilities of exposure. The fraction of Polluted beachline is defined as the fraction of sampling locations in the CBSA that exceeds the indicated pollution threshold, i.e., out of the 10 sampling locations in this CBSA, three of them are deemed polluted, and then the fraction of polluted beachline is 0.3 or 30%. Under the GM (STV) threshold, the probability of someone being exposed to polluted coastal water is 12% (7%). This is consistent with the estimates in the literature of [17]. Figure 2 depicts the probability that a certain faction of local beachline is polluted and it shows the probability distribution of the fraction of polluted local beachlines using both the STV and GM measures. 

#### 2.1.2. Current Population Survey (CPS)

The 2004–2013 waves of CPS were used to measure the amount of sick leave. This study observed individuals between 18 and 65 years of age who have a valid report of their race, ethnicity, gender, employment status, and sick leave. Sick leave is a YES or NO to the question, in which participants were asked “Have you ever taken a sick day from a labor market activity during the week before the CPS interview?”, where a labor market activity is a combination of working full-time, working part-time, and job search. 

This study linked the pollution indicators that were derived from the CEDEN to individuals in the CPS using the month, the year, and the longitude and latitude of the sampling locations. The data were kept for people who live in the California CBSAs that have a beach within their borders with at least one sampling location that consistently gathered ENT density data. These individuals live in the following cities: Los Angeles, Long Beach, Santa Ana, Salinas, San Diego, Carlsbad, San Marcos, San Francisco, Oakland, Fremont, San Luis Obispo, Paso Robles, Santa Barbara, Santa Maria, Goleta, Santa Cruz, Watsonville, Santa Rosa, Petaluma, Oxnard, Thousand Oaks, and Ventura. This process left us with 501,110 individuals. 

### 2.2. Methods

This section introduced the empirical strategies, where the study took advantage of the arguably exogenous variation in the degree of pollution of the coastal residents’ local beach waters. The empirical model in this study is more sophisticated than the models that were adopted in related studies. As stated in the introduction, several papers examined the health effects of visiting polluted California beaches [17,18,19]. However, these studies did not estimate the pollution-to-sickness statistics, instead, these studies adopted the numbers that were generated in [20] or [21]. In comparison to Cabelli [20] and Kay et al. [21], the method used in this study is similar to theirs in many respects. First, following their methods, this model includes demographic and social economic controls that take the background rate of disease into account; second, this model reduces the bias that is introduced by day-to-day variations of pollutant density by using a monthly measure. However, this study takes one step further and it looks at a crucial outcome of the illness, which is sick leave from work. In addition, this study looks at the intent-to-treat (ITT) effect, rather than the treatment-on-the-treated (TOT) effect. The ITT effect includes all individuals of interest, regardless of the treatment that these individuals actually received. While, TOT is the effect of taking the experiment. Applying this concept to this case, the ITT effect would be the effect of water being polluted on everyone who might be going to the beach. These people could have seen the warnings and left or could have stayed, because these people were unaware of the pollution or simply did not care. The TOT effect would be the effect of exposure to polluted water versus not exposed. Typically, for evaluating the effect of a policy, the ITT estimator is more useful, because this estimator is more relatable to the real-life effect of the policy.

Concerns for endogeneity after controlling for the independent variables in this model are low for the following reasons. First, the identification strategy that is used in this model not only relies on the fecal matter density, but also on the pollution criteria that are set by the government. Individual behavior must be somehow guided by the degree of pollution on local beachline for an omitted variable bias to occur. This is very unlikely, as individuals cannot predict the water quality with sufficient accuracy for a number of reasons. Fecal pollution in coastal waters is barely noticeable or observable with the naked eye. This type of pollution varies drastically day to day and responds primarily to the upstream pollution conditions tens of miles away from the coast. Fecal pollution is also largely influenced by events that are unrelated to health, such as an upstream city’s garden irrigation schedule or a sewage treatment plant’s release schedule. Official reports of water quality are usually days, if not weeks, behind the calendar day. Hence, individuals cannot predict the water quality with a sufficient degree of accuracy.

Equation (1) shows the reduced-form relationship between pollution and the number of forgone work or job-search days. Below is the empirical model that was adopted. Equation (1) was estimated while using the STATA^®^ software.

(1) $$Sick\;Leave_{igt}=\theta Polluted_{gt}+X_{igt}\gamma +\;\delta_{t}+\delta_{g}+\delta_{g}\times t+\varepsilon_{igt}$$

Productivity loss is measured with ***Sick Leave***, which a dummy indicator of taking sick leave in the previous week for an individual (***i***) in CBSA (***g***) and survey month (***t***). ***Polluted*** is the fraction of beachline that violated CCR policy in the residents’ CBSA in the month of the survey. The primary definition of Polluted is determined by the monthly GM threshold of 35 cfu/100 mL that the California regulations recommend. First, the geometric mean of each month’s ENT levels was calculated using the daily ENT according to the policy recommendations. Subsequently, this monthly GM was evaluated against the California regulations’ monthly GM criteria of 35 cfu/100 mL to determine whether the sampling location was contaminated for that month. The average probability of contamination across sampling locations was calculated to represent the degree of pollution in local coastal water if multiple sampling locations were in the same CBSA. The monthly average probability of violating the STV of 104 cfu/100 mL is treated as a secondary measure and used in the robustness tests. 

The covariates in X include the demographic variables for individual i, which include race (black, white, and other), ethnicity (Hispanic and non-Hispanic), and gender fixed effects. X also includes the employment status, which is a determinant of health [28]. γ is a vector of coefficients that corresponds to each X. $\delta_{t}$, $\delta_{g}$, and $\delta_{g}*t$ are the year fixed effect, the CBSA fixed effect, and the CBSA-specific linear time trend, respectively. The CBSA fixed-effects capture the geographic differences in the economic environment, population density, the quality of beach water, and the average beach-going behavior among local residents. The CBSA fixed-effects also capture the differences in the operation efficiency across the different local health care agencies that are responsible for educating the local population in beach water safety and posting warning signs on the beach when the ENT level exceeds the threshold. The year fixed effects account for the sporadic events each year that cause changes in the disease occurrences and the yearly variation in beach water quality, such as that caused by population growth. The CBSA-specific time trends capture the changes over time, such as gradual decays of each CBSA’s amenities that regulate fecal matter exposure, or the increase in the effort to educate the residents of water quality by CBSA, etc. The parameter θ, after controlling for these fixed effects, is arguably unbiased. *ε* is the random error. The 2004–2011 monthly rainfall (measured in mm) was retrieved from the CDC Wonder, which was used in the robustness checks. 

This study considers θ as the lower-bound estimate of productivity losses due to fecal matter, because it is possible that some individuals would attend work, even when these individuals do not feel well, while others may have jobs with flexible hours that do not require taking sick leave. θ also does not distinguish among the channels through which fecal matter affects health. According to the F-diagram of [29], the pathways of fecal pathogens are transmitted to a new host, either directly or indirectly, through digestion, fluids, physical contact, flies, or field crops. In California, besides the direct contact that was evaluated, the only other likely channel is through flies and/or food. Contact with contaminated runoff water upstream was difficult, since most watershed drains were not accessible during the study period. Therefore, this study shows that the predominant reason for expose to coastal fecal contaminants would be direct physical contact with the coastal water.

## 3. Results

### 3.1. Average Productivity Loss when Pollution Exceeds the Threshold

Table 2 panel A reports the results while using the monthly GM criteria of >35 cfu/mL as the measure for pollution. The first column presents the simple bivariate relationship between beach pollution and sick leave. This regression only uses the observations from the months during which there was local beach pollution that was determined by the monthly GM threshold. Columns (ii) to (iv) of Table 2 panel A gradually add the control variables and fixed effects until the model reaches the preferred specification stated in Equation (1). The coefficient that was reported in Column (iv) indicates that, if 100% of the local beachline has a pollution level above the monthly GM threshold of 35 cfu/100 mL, sick leave rises by 0.9 percentage points. To put this number into perspective, if the CPS sample is representative of the entire Californian population, then the effect can be translated into 3.56 million sick leave days per year due to polluted recreational water exposure among all Californians. 

### 3.2. Robustness Tests

The results in panel A were replicated using an alternative STV measure in panel B of Table 2. Exceeding the STV threshold signals a very high ENT level of at least 104 cfu/100 mL. The STV measure is less reliable than the GM measure, because the STV measure is subject to a larger measurement error due to the sampling locations and/or the time of the day. Nevertheless, there is a similar but more severe health effect (1.8 percentage points) from exceeding the STV pollution threshold. However, the standard errors are also much larger due to the variation in the daily ENT measurements. 

Panel A of Table 2 columns (v) and (vi) perform the robustness test, which considers the additional effects of rain on both measures of beach water quality. Rainfall increases the chance of sickness and it also increases the chance of the previously accumulated pollutants in the watershed to being washed to the shore [30]. Therefore, to prevent this omitted variable bias, the study controls for rainfall in this robustness test. These columns are based on individuals surveyed from 2004 to 2011 due to the limited years of rainfall data. Consequently, column (v) replicates the same exercise in column (iv) using 2004–2011 samples and serves as a point of comparison for adding the rainfall as a control variable in the analysis. The size of the effect in column (v) remained identical to column (iv), even with fewer observation years. Column (vi) adds the rainfall control variable. Controlling for rainfall did reduce the coefficient from 0.9 to 0.6 percentage points, although the change is statistically insignificant. This is consistent with our hypothesis that controlling for rainfall reduces upward omitted variable bias and helps to more accurately identify the effect of water quality. The 0.6 percentage point estimate was adopted as the primary and conservative estimate of the productivity loss due to runoff water pollution. A similar test was performed for the STV regressions in the Panel B columns (v) and (vi).

### 3.3. Effectiveness of the Water Quality Criteria

**Figure 3:**
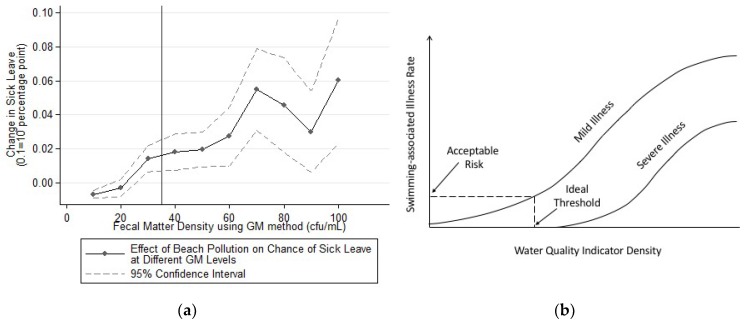
Effect of Exposure to Fecal-contaminated Coastal Water by Different Monthly Pollution Level. (**a**) The impact of having different pollution levels. The vertical line corresponds to the current CA monthly pollution criterium, which is GM level higher than 35 cfu/100 mL. (**b**) The ideal criteria suggested by Cabelli [20] based on a theoretical discussion of cost-benefit analysis.

This section explores the effectiveness of the criteria from two aspects: whether people comply with warning signs and whether the monthly/daily thresholds are sufficiently low to be at safe levels. Figure 3 shows the effect of changing the GM criteria on productivity loss. Figure 3a graphically illustrates the size of the effect according to the pollution level. Gradually increasing the monthly GM in Equation (1) from 10 to 110 in 10 cfu/mL intervals produces these estimates. This figure aims to inform the choice of a proper GM criterium for beach pollution. Each point is a separate regression that evaluates the increase of sick leave if the local beachline shifted from no pollution to polluted with fecal matter density near the said GM level (criterium-10 to criterium to be precise). For example, the value that was plotted for GM of 20 cfu/mL is the estimated effect on sick leave had the beach pollution level been at 10–20 cfu/mL. Equation (1) is used to estimate these effects. Figure 3b shows the equivalent theoretical deduction of the water pollution-sickness relationship and the ideal theoretical threshold proposed in the benchmark-setting work of Cabelli [20]. This paper is the first to show the empirical curve of Cabelli’s theoretical deduction.

Surprisingly, comparing the two figures shows that the pollution thresholds were set at a close-to-ideal level, and these thresholds should be reasonably safe. The theoretical prediction of the pollution-illness curve matches well with the shape of the empirical curve shown in Figure 3a. The ideal policy threshold should be strategically placed before the exponential growth segment of the curve for mild illnesses, which is almost precisely where 35 cfu/mL is on Figure 3a.

The lack of compliance with the policy is also what is surprising. Perfect compliance to the policy would imply that, whenever pollution exceeds the monthly GM threshold, a warning sign is posted, and everyone would follow the sign. The coefficient to the right of the threshold should be zero, because no one would have been exposed to polluted water. One would expect Figure 3a to display a sharp drop in the size of the coefficient immediately to the left of the threshold, even with imperfect compliance. However, there is not any observable discontinuity at the threshold.

**Figure 4:**
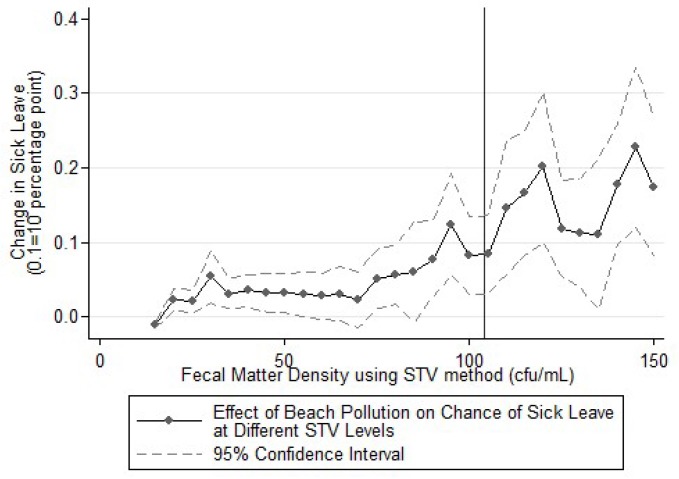
Effect of Exposure to Fecal-contaminated Coastal Water by Different Daily Pollution Level. The vertical line corresponds to the current CA daily pollution criterium, which is Statistical Threshold Value (STV) level higher than 104 cfu/100 mL.

To further test whether compliance is improved with the high STV threshold, a similar exercise was performed using the various levels of STV in Figure 4. This figure aims to inform the choice of a proper STV criterium for beach pollution. Each point is a separate regression that evaluates the increase of sick leave if the local beachline went from no pollution to polluted with fecal matter density near the said STV level (criterium-5 to criterium to be precise). For example, the value that is plotted for STV of 50 cfu/mL is the estimated effect on sick leave if the beach pollution level been at 45–50 cfu/mL. Equation (1) is used to estimate these effects. No obvious trend break is noticeable near the 104 cfu/100 mL thresholds either. 

## 4. Discussion

This study estimated the 3.56 million sick leave days per year due to polluted recreational water exposure among all Californians. This number is generally consistent with the GI infection predictions for Orange County’s Newport and Huntington Beaches based on historical pollution-to-sickness estimates [12]. The two beaches were predicted to generate an average of 36,778 GI episodes per year and approximately 38,000 more other illness episodes per year, which included respiratory, eye, and ear infections [19]. It is important to note that beach-pollution induced illness is not the most prominent reason for sick leave. Therefore, this study explains approximately 0.6 percent of all sick leave per year according to the r-squared statistics of the preferred specification. Albeit small, this fraction of sick leave is the result of beach water pollution. Additionally, the small fraction equates to a large number of sick leave in total, which should not be taken lightly by policymakers.

Based on this study, the importance of the AB 411 to public health should not be underestimated, but it is important to note that these GM and STV criteria came into being rather haphazardly, for the purpose of fully understanding this policy. These criteria were converted from the coliform thresholds that were released by National Technical Advisory Committee in 1968 [24]. These coliform thresholds were obtained from a study by the U.S. Public Health Service, which found the threshold of sickness-inducing coliform density to be 400 fecal coliforms per 100 mL [31]. These results are referred to by the USEPA as “far from definitive”, and the agency arbitrarily cut this density in half to 200 fecal coliforms per 100 mL in the 1960s [25]. Cabelli [20] and Dufour [32] later found that ENT density is a better predictor of GI illnesses when compared to coliform. In 1986, an unproven formula was implemented by the USEPA [25] to convert coliform into ENT standards. It is easy to see that various steps of the generation of the ENT criteria are debatable and the thresholds are not based on empirical research regarding ENT. Therefore, the policy calls for further tests on its effectiveness.

The effectiveness of the water quality criteria was assessed by two tests in this study. The first test is to determine the safeness of the monthly/daily water quality thresholds. These tests are important for a number of reasons. First, the only enforcement method is posting warning signs or closure signs. It would be beneficial for policymakers to test whether this is sufficient for preventing exposure to contaminated coastal water. Second, the California regulations have set the monthly GM pollution threshold for warning the public at 35 cfu/100 mL of ENT. This ENT standard is an arbitrary conversion of a debatable threshold measured using the fecal coliform density. Therefore, it is useful to see whether an empirical test that is based on ENT renders the ideal disease-prevention effect as is theoretically deduced. Surprisingly, the results indicate that the somewhat outdated pollution thresholds are reasonably safe and appropriate. The empirical pollution-to-sickness curve matches well with the theory that was proposed by Cabelli [20]. The theoretically optimal threshold should be the start of the segment of accelerated growth in the pollution-to-sickness curve. Both the GM and STV thresholds precede the accelerated growth segment of their corresponding curves based on the shape of the empirical curve estimated in this study.

The second test focused on whether the recreators comply with the warning signs. As previous discussions suggested, our study should expect a significant decrease in the size of the effect when the thresholds were exceeded if compliance to the policy is high. Figure 3 and Figure 4 showed no noticeable trend breaks near the GM or STV thresholds. The lack of such trend breaks indicates inadequate compliance to the existing policy being noticed using both monthly and daily water quality thresholds. If the current policy was strictly enforced, there would be a significant reduction in sick leaves and a corresponding gain in productivity. This finding implies that the warning signs were quite ineffective in preventing exposure to contaminated coastal water, and more effective measures are necessary. 

The lack of enforcement of the policy is an alternative explanation for the lack of compliance. Brinks et al. [17] mentioned that the number of times a warning or a closure sign is posted on the beaches is much lower than the 12% estimated with the data. Meanwhile, it is worth noting that the enforcement of policies is often a clouded area with a lot of stakeholders weighing in to achieve the best balance that benefits the stakeholders in a fair manner. Preventing visitors from going certainly faces backlash from the beach-loving communities and beach cities, whose economy heavily depends on beach visitors. In fact, some beach lovers are willing to take the risk of going to “polluted” beach, despite the high-bacteria level warning. Therefore, the reality is that a beach is often closed when there is a sewage overflow or spill upstream from the beach, which is a real more serious risk, but it is not based on the ENT level of its water. 

## 5. Conclusions

This study assesses the existing California Code of Regulations that controls the beach water quality monitoring for fecal contamination and determines the productivity losses due to exposure to beach fecal contamination. The hypothesis is that successful regulation would lead to no productivity losses. The study found a 0.6 percentage point increase in sick leave when the GM criterium is exceeded, which translates into 3.56 million sick leave days per year in coastal California. Furthermore, the regression model that was used is robust to alternative specifications. The results reveal that the beach water quality criteria are set at reasonably safe levels. However, the enforcement of the regulations needs to be strengthened to prevent visitors from going to polluted beaches. Public awareness of beach pollution effects on health should also be increased to prevent illness.

## Figures and Tables

**Table 1:** Summary Statistics. (Please add the column heading.)

Variable	Observations ^1^	Mean	Standard Deviation	Min	Max
**CPS Individual Survey**					
Year	522,080	2008.70	2.81	2004	2013
Month	522,080	6.67	3.40	1	12
Age	522,080	39.91	13.21	18	65
White	522,080	0.75	0.44	0.00	1.00
Black	522,080	0.07	0.25	0.00	1.00
Hispanic	522,080	0.36	0.48	0.00	1.00
Gender	522,080	0.50	0.50	0.00	1.00
Sick Leave	522,080	0.02	0.13	0.00	1.00
CDC WONDER Weather Dataset Rainfall ^2^	409,923	1.11	1.78	0.00	17.49
Polluted Beachline by CBSA and Month ^3^					
Determined by GM Criteria:					
>35 cfu/100 mL	1202	0.12	0.18	0	1
>50 cfu/100 mL	1202	0.08	0.15	0	1
35–50 cfu/100 mL	1202	0.04	0.09	0	1
Determined by STV Criteria:					
>104 cfu/100 mL	1202	0.07	0.12	0	1

^1^ The Current Population Survey (CPS) data were on the individual level. The survey answers from 522,080 individuals were used in this study. The data on rainfall were unavailable in 2012–2013, which led to a smaller number of individuals in these years, or a total of 409,923. The pollutant dataset was organized based on CBSA at a monthly scale. There were 1202 CBSA-by-month pollution measures utilized in this study. ^2^ Rainfall by month and county were measured in mm and obtained from CDC Wonder Weather Dataset. ^3^ Polluted beachline is defined as the fraction of sampling locations in the CBSA that exceeds the indicated pollution threshold.

**Table 2:** The Effect of Exposure to Fecal-contaminated Coastal Water on Taking Sick Leave Using Alternative Specification and Control for Rain Fall.

Independent Variable	(i)	(ii)	(iii)	(iv)	(v)	(vi)
*Panel A: GM threshold* % Beachline Polluted	0.005 ***	0.009 ***	0.009 ***	0.009 ***	0.009 ***	0.006 **
	(0.001)	(0.001)	(0.001)	(0.001)	(0.002)	(0.003)
R^2^	0.001	0.001	0.001	0.006	0.005	0.005
*Panel B: STV threshold* % Beachline Polluted	0.009 ***	0.020 ***	0.018 ***	0.018 ***	0.018 ***	0.014 ***
	(0.002)	(0.003)	(0.003)	(0.003)	(0.003)	(0.005)
R^2^	0.001	0.001	0.001	0.006	0.005	0.005
Year FE		Y	Y	Y	Y	Y
CBSA FE		Y	Y	Y	Y	Y
CBSA-specific Time Trend			Y	Y	Y	Y
Demographics				Y	Y	Y
Rainfall					Y	Y
Years of Data Included	2004–2013	2004–2013	2004–2013	2004–2013	2004–2011	2004–2011

Columns (i) to (iii) gradually adds control variables to show consistency of our analysis. The control variables in column (iv) is the same as specified in Equation (1). The included control variables for each column are indicated on the bottom panel of the table. Y indicates that the corresponding control variable is included. White-robust standard errors are used and reported in the parentheses. ** significant at 5 percent; *** significant at 1 percent.
